# Exercise in pregnancy: 1-year and 7-year follow-ups of mothers and offspring after a randomized controlled trial

**DOI:** 10.1038/s41598-018-30925-5

**Published:** 2018-08-27

**Authors:** Valentina Chiavaroli, Sarah A. Hopkins, José G. B. Derraik, Janene B. Biggs, Raquel O. Rodrigues, Christine H. Brennan, Sumudu N. Seneviratne, Chelsea Higgins, James C. Baldi, Lesley M. E. McCowan, Wayne S. Cutfield, Paul L. Hofman

**Affiliations:** 1 Liggins Institute, University of Auckland, Auckland, New Zealand; 2 A Better Start – National Science Challenge, University of Auckland, Auckland, New Zealand; 3 Department of Women’s and Children’s Health, Uppsala University, Uppsala, Sweden; 4 Department of Paediatrics, Faculty of Medicine, University of Colombo, Colombo, Sri Lanka; 5 Department of Medicine, Dunedin School of Medicine, University of Otago, Dunedin, New Zealand; 6 Department of Obstetrics and Gynaecology, Faculty of Medical and Health Sciences, University of Auckland, Auckland, New Zealand

## Abstract

There are limited data on long-term outcomes of mothers or their offspring following exercise interventions during pregnancy. We assessed long-term effects of an exercise intervention (home-based stationary cycling) between 20–36 weeks of gestation on anthropometry and body composition in mothers and offspring after 1 and 7 years. 84 women were randomised to intervention or usual activity, with follow-up data available for 61 mother-child pairs (38 exercisers) at 1 year and 57 (33 exercisers) at 7 years. At 1 year, there were no observed differences in measured outcomes between mothers and offspring in the two groups. At the 7-year follow-up, mothers were mostly similar, except that exercisers had lower systolic blood pressure (−6.2 mmHg; p = 0.049). However, offspring of mothers who exercised during pregnancy had increased total body fat (+3.2%; p = 0.034) and greater abdominal (+4.1% android fat; p = 0.040) and gynoid (+3.5% gynoid fat; p = 0.042) adiposity compared with controls. Exercise interventions beginning during pregnancy may be beneficial to long-term maternal health. However, the initiation of exercise during pregnancy amongst sedentary mothers may be associated with adverse effects in the offspring during childhood. Larger follow-up studies are required to investigate long-term effects of exercise in pregnancy.

## Introduction

Pregnancy is a vulnerable period, when perturbations in the *in utero* environment may affect fetal metabolism and growth. Indeed, there is a large body of evidence indicating that intrauterine disturbances (e.g. exposure to maternal obesity) are associated with long-term adverse health outcomes for the offspring, such as increased risk of obesity, hypertension, and coronary artery disease^1,2^. Thus, a healthy lifestyle (i.e. balanced diet and regular physical activity) is recommended during pregnancy to promote healthy fetal growth and development. Namely, physical activity is advised to most woman entering pregnancy, and there is increasing evidence that, in absence of obstetric or medical contraindications, regular moderate-intensity aerobic exercise (e.g. daily walking or stationary cycling >30 minutes/day) is beneficial to maternal health^3–5^. Although the evidence is limited^3,4^, regular exercise has been associated with reduced risk of excessive weight gain during pregnancy^6^, gestational diabetes^7^, and hypertension^8^. In contrast, long-term maternal outcomes after exercise throughout pregnancy have been poorly investigated, and the available studies have focused mainly on the immediate postpartum period^9,10^. Since pregnancy has been indicated as a period when women are at risk for the development of obesity^11^, it is necessary to establish whether exercise during pregnancy improves long-term maternal health outcomes.

Exercise in pregnancy has the potential to influence the *in utero* environment and feto-placental growth through the modulation of placental blood flow, nutrient partitioning and oxygen/nutrient delivery to the fetus^12^. However, the data on the effects of maternal exercise on offspring outcomes (e.g. birth weight and adiposity) are conflicting. Some studies have demonstrated no effect of maternal physical activity on birth weight^13,14^, while others have found increased birth weight in response to low- or moderate-intensity exercise started during pregnancy^12,15^. In contrast, prospective cohort studies have suggested that previously active women who maintain a high volume of exercise into the third trimester tend to deliver infants weighing 200–400 g less than non-exercise controls^12,16,17^. In this respect, Clapp *et al*. suggested that the prescription of exercise (type, duration, frequency, and intensity) and the stage of pregnancy have varying effects on both size and body composition at birth^12^. For instance, a low volume of exercise in mid and late pregnancy appears to stimulate fetal growth, regardless of exercise performance in early pregnancy^12,15^, while a high volume of weight-bearing exercise in later gestation restricts fetal fat deposition^12^. This probably reflects alterations in insulin sensitivity and nutrient partitioning in the second half of pregnancy, and may also explain some of the discrepancies found among studies. However, overall the available evidence is inconclusive as to whether antenatal exercise exhibits beneficial or detrimental effects on fetal growth.

To date it seems that only one group has investigated in a prospective case-control study the impact of exercise in pregnancy on the offspring in childhood^18^, showing a leaner phenotype at age 5 years in children exposed to maternal physical activity compared to controls. Confirmatory studies and longer follow-up of offspring exposed to antenatal exercise, especially of participants in randomized controlled trials (RCTs), is essential to establish long-term effects and whether it is actually beneficial. We have previously shown in a RCT that moderate-intensity exercise over the second half of gestation in healthy nulliparous women led to an average birth weight reduction of approximately 250 g^19^. Although no increased risk of preterm or small-for-gestational-age birth was noted in the exercise arm of our study, it was important to evaluate if the birth weight reduction in the exercise group affected long-term metabolic outcomes. Thus, in the present study we aimed to assess the effects of exercise during pregnancy on metabolism and body composition in mothers and offspring at approximately 1 year and 7 years after birth.

## Subjects and Methods

### Ethics approval

Ethics approval for this study was provided by the Multiregion Ethics Committee (Ministry of Health, New Zealand). Written informed consent was obtained from parents or guardians, as well as verbal or written consent from each child as was appropriate to their age. This study was performed in accordance with all appropriate institutional and international guidelines and regulations for medical research, in line with the principles of the Declaration of Helsinki.

### Participants

This study involved the follow-up of mothers and their offspring who participated in a community-based RCT of exercise in pregnancy^19^. The original study included 84 healthy nulliparous women with a singleton pregnancy of less than 20 weeks of gestation and who were relatively sedentary. All participants were randomly assigned to exercise (n = 47) or control (n = 37) groups.

### Exercise protocol

The study protocol recommended that regular exercise was started from 20 weeks of gestation and maintained until at least 36 weeks. Participants went through a familiarisation period (gestational weeks 20 to 27) designed to encourage adjustment to regular exercise and to build exercise tolerance. Exercise prescription during these initial 8 weeks was variable and dependent upon individual needs. During the maintenance period (weeks 28 to 35) participants in the exercise group were asked to complete 5 sessions of 40 minutes of aerobic exercise per week, at an intensity corresponding to 65% of their baseline predicted VO_2max_. Between weeks 36 and 40 exercise tolerance was variable, and participants were asked to continue exercising as much as possible subject to their perceived exercise capacity.

Each exercise session began with a low-intensity warm-up of around 5 minutes duration (either on the stationary cycle ergometer or walking), after which time each participant completed a period of prescribed exercise of up to 40 minutes duration. Each cycle ergometer was equipped with a manually controlled resistance wheel with 8 different resistance settings. Exercise intensities were prescribed in two ways and recorded in a prescription chart for the participants to follow: a) cycling specific workloads, including resistance level and a range for cycling speed in revolutions per minute; and/or b) a prescribed heart rate range (beats per minute). Participants were then free to adjust their workload within and between sessions as necessary, but were asked to record any changes in intensity in their training diary.

Control participants were asked to continue their normal daily activities for the duration of their pregnancy.

### Clinical assessment at 1 year

Clinical assessments were carried out at the Maurice & Agnes Paykel Clinical Research Unit (Liggins Institute, University of Auckland). Children underwent anthropometric measurements, including weight, length, abdominal circumference, and hip circumference. Maternal height and weight were measured, and body mass index (BMI) was calculated. Paternal height was obtained during the trial, and mid-parental height standard deviation score (MPHSDS) was calculated using standard formulae^20^. Children’s height, weight and BMI were transformed into SDS^21,22^. Children’s height SDS were then individually adjusted for their genetic potential (parents’ heights), using the formula: [child’s height SDS – MPHSDS].

### Clinical assessment at 7 years

All children underwent a physical examination, including anthropometric measurements and pubertal staging (all participants were prepubertal corresponding to Tanner stage 1). Standing height was measured to the nearest mm using a Harpenden stadiometer.

Maternal and children’s body composition was assessed using whole-body dual-energy x-ray absorptiometry (DXA Lunar Prodigy 2000; General Electric, Madison, WI, USA). The DXA-derived parameters of interest were total body fat, total lean mass, and android fat to gynoid fat ratio (an indicator of abdominal adiposity).

Maternal and children’s resting systolic and diastolic blood pressures were measured on the non-dominant arm, after five minutes of rest, and with an appropriately-sized cuff, using the same calibrated and accurately-maintained sphygmomanometer (Dinamap ProCare 100; GE Healthcare, Chalfont St Giles, UK). Following an overnight fast, morning blood samples were drawn to measure serum concentrations of glucose, insulin, total cholesterol, high-density lipoprotein cholesterol (HDL-C), low-density lipoprotein cholesterol (LDL-C), and triglycerides. Insulin sensitivity was evaluated using the homeostasis model assessment of insulin resistance (HOMA-IR).

Food diaries were collected for both mothers and children, and nutritional intake was estimated using standard household measures and food labels where appropriate. Records were entered into Foodworks software (v5.0, Xyris Software, Brisbane, Australia) by a trained investigator. Maternal physical activity levels were assessed using a physical activity questionnaire, covering four domains of physical activity: work-related, transportation, housework/gardening, and leisure time^23^. Data on children’s weekly physical activity levels were provided by the mothers via a questionnaire.

### Outcomes

The primary outcome of the original trial was birth weight^19^. In this follow-up study, the outcomes of interest were anthropometry (weight, height, and BMI), body composition (total body fat, android fat, gynoid fat, and android fat to gynoid fat ratio), and metabolism (blood pressure, glucose homeostasis, and lipid profile) in mothers and offspring at approximately 1 year and 7 years after birth.

### Assays

Insulin concentrations were measured using a COBAS e411 autoanalyser (Hitachi High Technologies Corporation, Tokyo, Japan) by electrochemiluminescence immunoassay, with an interassay coefficient of variation (CV) of 2.4%. Glucose, triglycerides, total cholesterol and HDL-C concentrations were measured on a COBAS c311 autoanalyser (Hitachi) by enzymatic colorimetric assay (Roche, Mannheim, Germany), with interassay CVs of 2.0, 1.4, 2.8, and 1.9% respectively.

### Statistical analysis

Demographic characteristics between groups were compared with one-way ANOVA, Chi-square tests, and Fisher’s exact tests. Outcomes in both groups were compared using general linear regression models, accounting for important confounding factors. Models examining maternal one-year outcomes on anthropometry and body composition were adjusted for BMI, ethnicity, and age at trial baseline, while blood pressure data were adjusted for ethnicity, height, and age. For maternal 7-year outcomes on anthropometry and body composition, models were adjusted for BMI at trial baseline, ethnicity, current age, and number of pregnancies since the trial; for blood pressure, models were adjusted for ethnicity, height, age, and number of pregnancies. To assess offspring outcomes, models accounted for maternal age and ethnicity, child’s sex and age, as well as one of the following: i) maternal BMI at trial baseline for weight and body composition; ii) MPHSDS for height; and iii) child’s height for blood pressure. The interaction between group allocation and sex was evaluated for all parameters, and where a significant interaction was observed (p < 0.05) data were analysed separately for boys and girls to examine sex-specific effects.

Where appropriate, data were log-transformed to approximate a normal distribution. Statistical analyses were carried out in SAS version 9.4 (SAS Institute Inc. Cary NC, USA) and Minitab v.16 (Pennsylvania State University, State College, PA, USA). All statistical tests were two-tailed and maintained at a 5% significance level, without adjustment for multiple comparisons. Outcome data are presented as model-adjusted means (estimated marginal means adjusted for the confounding factors in the models), with associated 95% confidence intervals.

## Results

**Figure 1:**
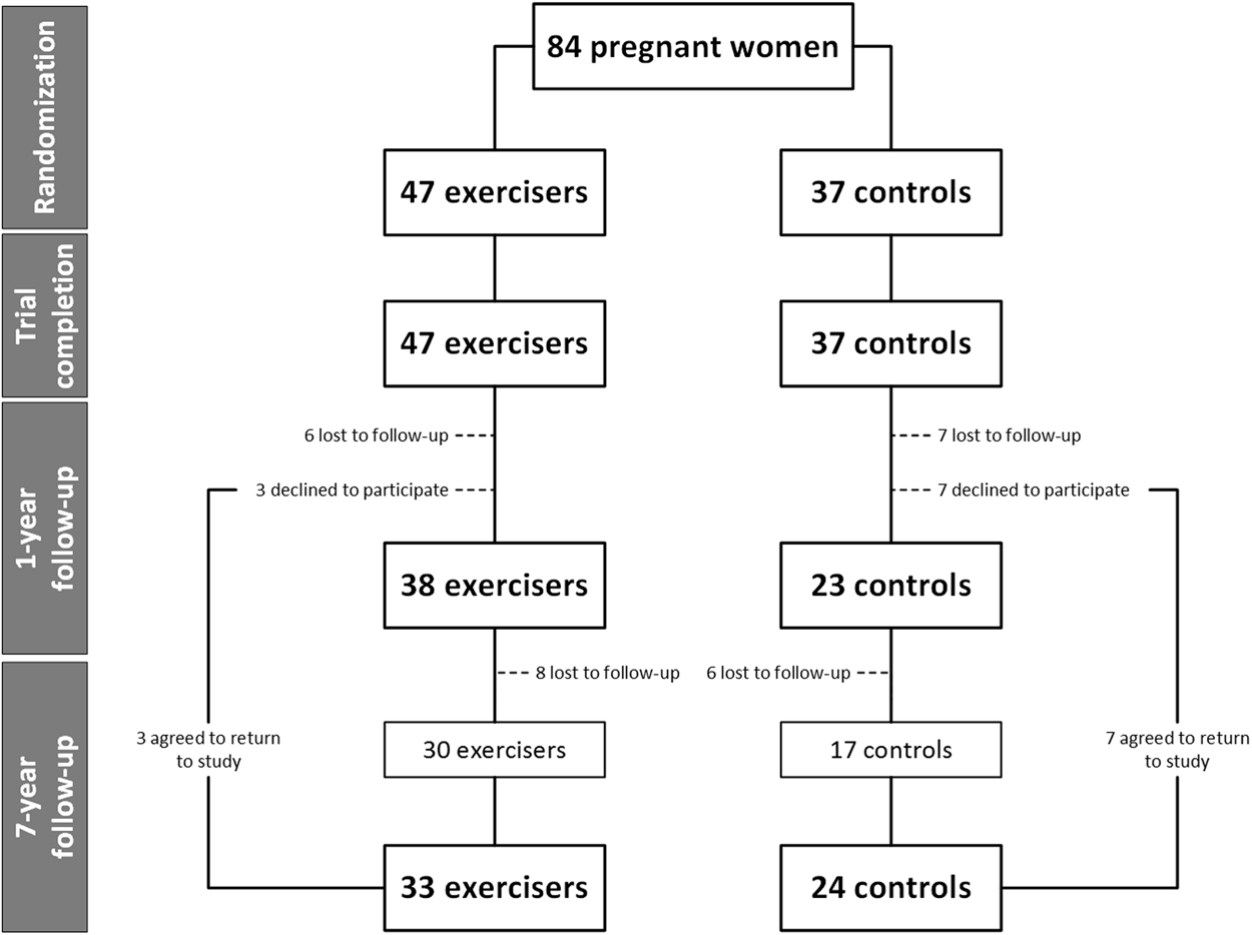
Summary of study recruitment and follow-up. Note that numbers at the 1-year follow-up represent the number of children assessed, as 18 mothers were not assessed (including 10 mothers who were pregnant again at the time of assessment).

Of the initial 84 women and offspring who participated in the original trial, 1-year follow-up data were available on 43 mothers (30 exercisers, 13 controls) and 61 children (38 exercisers, 23 controls), while 7-year follow-up data were available on 57 mothers (33 exercisers, 24 controls) and 57 children (Fig. 1). The main reasons for loss to follow-up were living outside the Auckland region or being uncontactable. The proportion of participants lost to follow-up in exercise and control groups was similar throughout the study period (Fig. 1). In addition, maternal parameters at study entry were similar in the follow-up and lost groups, as were most offspring parameters (data not shown).

### Maternal outcomes at 1- and 7-year follow-ups

At 1-year follow-up there were no observed differences in any of the assessed parameters between mothers in the exercise and control groups (Table 1).

**Table 1:** Demography, and anthropometric and metabolic outcomes at the 1-year and 7-year follow-up visits in control mothers and those who exercised in pregnancy.

		1-year follow-up			7-year follow-up		
		Control	Exercise	p-value	Control	Exercise	p-value
n		13	30		24	33	
Demography	Age (years)	31.6 ± 3.5	33.1 ± 3.0	0.18	37.9 ± 3.6	39.5 ± 3.4	0.07
	Ethnicity (European)	77%	93%	0.15	84%	89%	0.52
Anthropometry	BMI at baseline (kg/m^2^)	26.0 ± 2.5	25.3 ± 3.5	0.57	25.5 ± 3.1	25.3 ± 4.2	0.85
	Height (cm)	166.4 ± 7.4	165.0 ± 6.7	0.56	167.7 ± 7.2	165.6 ± 6.9	0.26
	Weight (kg)	66.9 (64.7, 69.2)	65.7 (64.3, 67.2)	0.38	69.7 (67.0, 72.3)	67.6 (65.3, 69.8)	0.24
	BMI (kg/m^2^)	24.4 (23.6, 25.3)	24.0 (23.5, 24.5)	0.39	25.1 (24.1, 26.1)	24.3 (23.5, 25.1)	0.21
Body composition	Total body fat (%)	33.3 (29.4, 37.2)	31.8 (29.1, 34.4)	0.51	33.5 (30.5, 36.4)	32.1 (29.4, 34.8)	0.51
Blood pressure	Systolic (mmHg)	114.3 (105.1, 123.4)	113.0 (107.0, 119.0)	0.81	111.0 (106.4, 115.6)	104.8 (100.9, 108.7)	**0.049**
	Diastolic (mmHg)	70.5 (63.8, 77.2)	68.0 (63.5, 72.4)	0.53	67.3 (63.7, 70.9)	65.0 (61.9, 68.1)	0.35

Age, height, and BMI at trial baseline data are means ± standard deviations; 1-year data on anthropometry (except height) and body composition are means and 95% confidence intervals (CI) adjusted for body mass index (BMI), ethnicity, and age at trial baseline, while blood pressure data were adjusted for ethnicity, height, and age; 7-year data on anthropometry (except height) and body composition are means and 95% CI adjusted for BMI at trial baseline, ethnicity, current age, and number of pregnancies since the trial, while blood pressure data were adjusted for ethnicity, height, age, and number of pregnancies.

At 7 years, women in the two groups were mostly similar, except that mothers who exercised in pregnancy had systolic blood pressure that was 6.2 mmHg lower (p = 0.049) than control mothers (Table 1). Dietary intake and physical activity levels were similar in the two groups (Supplemental Table 1).

**Figure 2:**
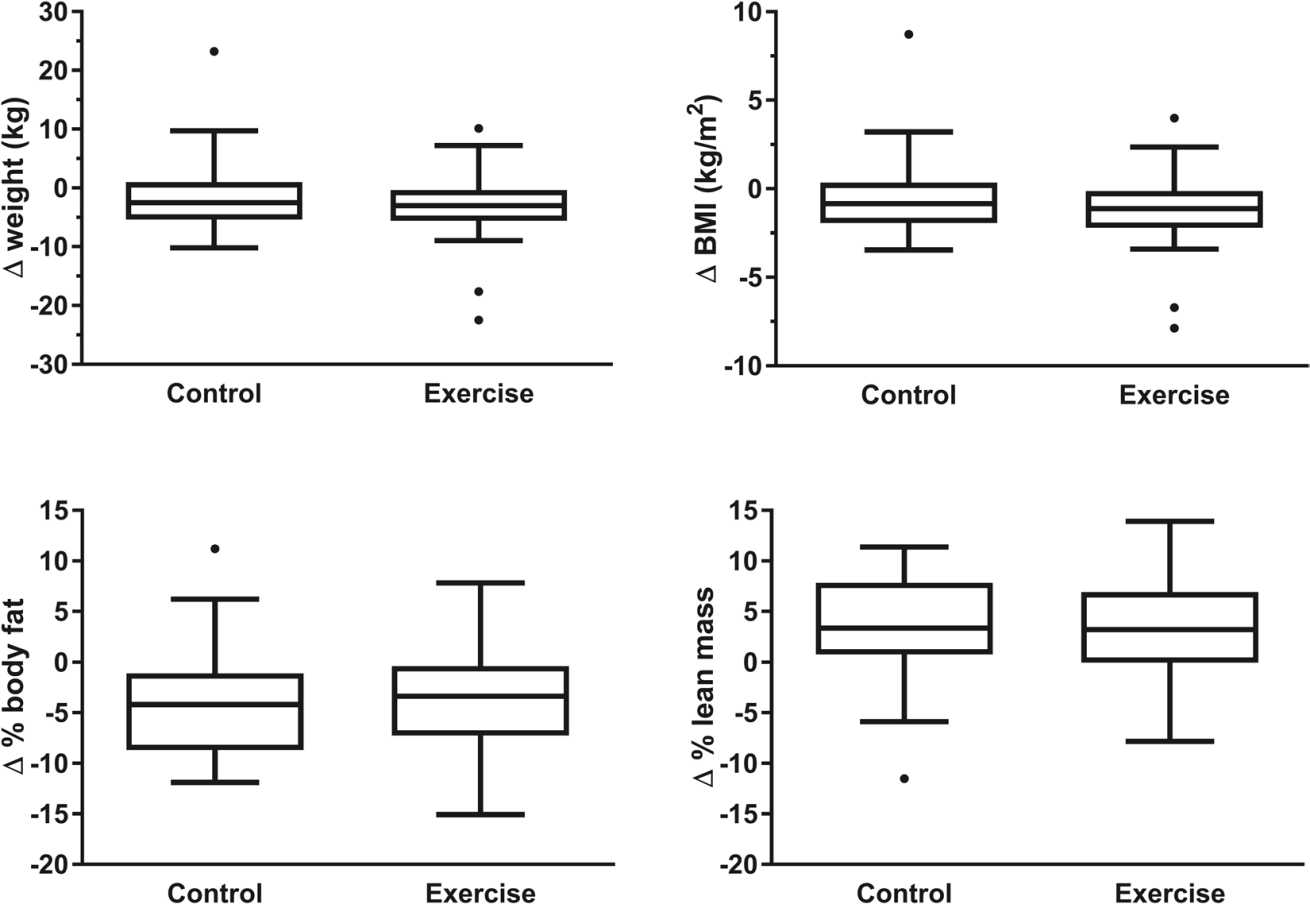
Changes in anthropometry and body composition to follow-up at 7 years within control mothers (n = 24) and those who exercised in pregnancy (n = 33). Weight and body mass index (BMI) differences relate to pre-pregnancy values, while body composition changes are compared to measurements taken 2 weeks after delivery. Outliers have been determined using Tukey’s method^35^, with inner fences equal to [Q1 – (1.5 * IQR)], where Q1 is quartile 1 and IQR is the interquartile range.

**Figure 3:**
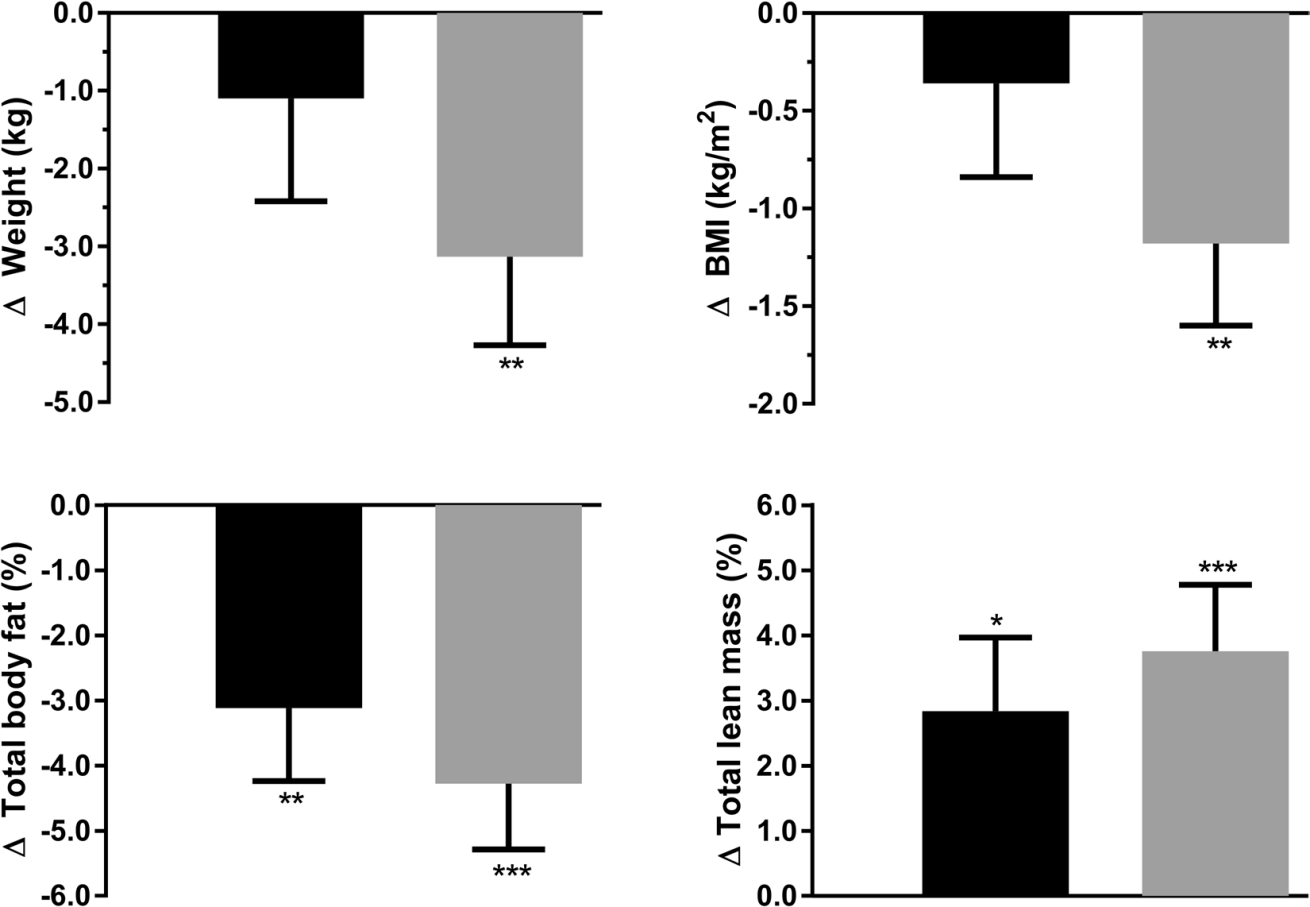
Differences in anthropometry and body composition to follow-up at 7 years within control mothers (black) and those who exercised in pregnancy (grey). Weight and body mass index (BMI) differences relate to pre-pregnancy values, while body composition changes are compared to measurements taken 2 weeks after delivery. Data are expressed as the mean change from baseline (Δ) and standard error, adjusted for BMI at baseline, current age, and number of pregnancies since the trial. *p < 0.05, **p < 0.01, and ***p < 0.001 vs baseline. There were no statistically significant differences between groups.

Figure 2 illustrates the distribution of changes in maternal anthropometry and body composition between the trial and the 7-year follow-up. When maternal outcomes were assessed within each group from early pregnancy to follow-up, adjusted analyses showed a significant reduction in weight (−3.1 kg; p = 0.008) and BMI (−1.18 kg/m^2^; p = 0.007) within women who exercised in pregnancy (Fig. 3). There were also reductions in percentage body fat in both exercise (−4.3%; p = 0.0001) and control groups (−3.1%; p = 0.008) from 2 weeks after delivery, with corresponding increases in percentage lean mass (Fig. 3). However, the magnitude of these changes was not significantly different between groups.

### Offspring outcomes at 1- and 7-year follow-ups

Children were assessed at a mean age of 1.1 years (range 1.0–1.3 years). As previously reported^19^, children in the exercise group were 0.47 SDS lighter (p = 0.013) than controls at birth. However, at the 1-year follow-up, there were no differences in anthropometry between exercise and control groups (Table 2).

**Table 2:** Demography, and anthropometric and metabolic outcomes at 1-year follow-up in the offspring of control mothers and those who exercised in pregnancy.

		Control	Exercise	p-value
n		23	38	
Demography	Age (years)	1.06 ± 0.06	1.05 ± 0.05	0.79
	Sex ratio (males)	43%	58%	0.27
	Ethnicity (European)	91%	74%	0.08
Anthropometry	Weight (kg)	9.89 (9.5, 10.3)	9.90 (9.6, 10.2)	0.95
	Weight SDS	−0.20 (−0.63, 0.22)	−0.16 (−0.50, 0.17)	0.88
	Height (cm)	75.7 (74.6, 76.8)	76.3 (75.4, 77.1)	0.42
	Height SDS – MPHSDS	−0.35 (−0.80, 0.10)	−0.02 (−0.36, 0.32)	0.25
	BMI SDS	−0.26 (−0.70, 0.18)	−0.38 (−0.73, −0.04)	0.66
	Waist circumference (cm)	46.9 (45.7, 48.1)	46.1 (45.1, 47.0)	0.28
	Hip circumference (cm)	43.2 (42.0, 44.5)	42.8 (41.9, 43.9)	0.64
	Waist to hip ratio	1.09 (20.3, 22.1)	1.09 (19.6, 21.1)	0.78

BMI, body mass index; BMI SDS, body mass index standard deviation score; MPHSDS, mid-parental height standard deviation score; SDS, standard deviation score.

Age data are means ± standard deviations; other data are means and 95% confidence intervals adjusted for confounding factors in multivariable models, namely maternal age and ethnicity, child’s sex and age, as well as one of the following: (i) MPHSDS for length; and (ii) maternal BMI at trial baseline for weight and other body proportions.

Children were subsequently re-assessed at a mean age of 7.6 years (range 6.1–8.9 years). Exercise and control children remained anthropometrically similar (Table 3). However, the offspring of mothers who exercised during pregnancy had increased total body fat (+3.2%; 0.034), and greater abdominal (+4.1% android fat; p = 0.040) and gynoid (+3.5% gynoid fat; p = 0.042) adiposity than control children (Table 3). There were no significant differences in blood pressure. Glucose levels were slightly lower in children born to mothers who exercised during pregnancy than children born to control mothers (4.85 *vs* 5.16 mmol/L; p = 0.037), without differences in insulin and HOMA-IR levels (Table 3). There were no differences in lipid profiles (Table 3).

**Table 3:** Demography, and anthropometric and metabolic outcomes at 7-year follow-up in children born to mother who exercised in pregnancy or to control mothers.

		Control	Exercise	p-value
n		24	33	
Demography	Age (years)	7.7 ± 0.7	7.6 ± 0.8	0.69
	Sex ratio (males)	42%	64%	0.42
	Ethnicity (European)	92%	73%	0.10
Anthropometry	Weight (kg)	26.9 (25.3, 28.4)	26.5 (25.4, 27.6)	0.68
	Height (cm)	129.6 (126.8, 132.4)	128.3 (126.3, 130.3)	0.40
	Height SDS – MPHSDS	0.47 (−0.07, 1.01)	0.24 (−0.13, 0.62)	0.44
	BMI SDS	0.14 (−0.26, 0.54)	0.12 (−0.16, 0.40)	0.92
Body composition	Total body fat (%)	15.3 (12.7, 18.0)	18.5 (16.6, 20.4)	**0**.**034**
	Android fat (%)	17.5 (13.9, 21.08)	21.6 (19.0, 24.2)	**0**.**040**
	Gynoid fat (%)	28.0 (24.9, 31.0)	31.4 (29.2, 33.7)	**0**.**042**
	Android fat to gynoid fat ratio	0.62 (0.54, 0.70)	0.67 (0.62, 0.73)	0.20
Blood pressure	Systolic (mmHg)	96.0 (89.7, 102.3)	101.8 (97.1, 106.5)	0.09
	Diastolic (mmHg)	58.0 (53.3, 62.8)	59.4 (55.9, 62.9)	0.60
Glucose homeostasis	Fasting glucose (mmol/L)	5.16 (4.90, 5.42)	4.85 (4.64, 5.06)	**0**.**037**
	Fasting insulin (mIU/L)	7.98 (5.36, 11.89)	6.40 (4.63, 8.85)	0.32
	HOMA-IR	1.82 (1.18, 2.81)	1.38 (0.97, 1.96)	0.25
Lipid profile	Total cholesterol (mmol/L)	5.21 (4.77, 5.65)	5.04 (4.68, 5.40)	0.49
	HDL-C (mmol/L)	1.71 (1.55, 1.87)	1.68 (1.55, 1.81)	0.69
	LDL-C (mmol/L)	3.31 (2.83, 3.79)	3.19 (2.80, 3.58)	0.67
	Triglycerides (mmol/L)	0.86 (0.69, 1.03)	0.83 (0.70, 0.96)	0.75

BMI, body mass index; BMI SDS, body mass index standard deviation score; HDL-C, high-density lipoprotein cholesterol; HOMA-IR, homeostasis model assessment of insulin resistance; LDL-C, low-density lipoprotein cholesterol; MPHSDS, mid-parental height standard deviation score; SDS, standard deviation score.

Age data are means ± standard deviations; other data are means and 95% confidence intervals adjusted for confounding factors in multivariable models, namely maternal age and ethnicity, child’s sex and age, as well as one of the following: (i) MPHSDS for height; (ii) maternal BMI at trial baseline for weight, BMI SDS, and body composition; and (iii) child’s height for blood pressure.

Dietary intake was similar in children born to mothers who exercised during pregnancy or to control mothers (Supplemental Table 2). Physical activity levels were also similar between groups (p = 0.33).

### Sex-specific effects

There was no evidence of a differential effect of maternal exercise during pregnancy amongst boys or girls at age one year (data not shown). At the 7-year follow-up, there was a significant interaction between trial allocation and sex for two parameters, but without statistically significant differences between groups (Supplementary Table 3).

## Discussion

In this follow-up study of nulliparous mothers and their offspring after a RCT, exercise during pregnancy may have resulted in a small benefit to maternal health. We observed lower systolic blood pressure after 7 years in those who exercised; further, while there was a reduction in adiposity in both groups of mothers, exercisers were also lighter and leaner compared to pre-pregnancy. Nonetheless, the data suggest that exercise in pregnancy, in mothers who were not previously physically active, may be associated with some subtle adverse consequences to the offspring in childhood.

A healthy lifestyle during pregnancy may influence later maternal body composition and reduce obesity risk, but few studies have prospectively assessed the long-term effects of exercise in pregnancy^9,10,18^. Phelan *et al*. showed that normal-weight and overweight/obese women whose intervention included low-intensity exercise during pregnancy (i.e. 30 minutes of walking most days of the week) retained less weight 12 months postpartum^10^. The same effect was observed in healthy nulliparous women who attended a RCT of prenatal dietary counselling and exercise sessions^24^. Similar evidence has been provided by a systematic review and meta-analysis, in which overweight/obese women exposed to physical activity plus diet showed greater weight reduction (−1.50 kg) than controls^25^. Importantly, only one study examined the effects of exercise in pregnancy on maternal outcomes beyond the first year after childbirth, reporting no differences in body composition between mothers who exercised versus those who did not at 5 years postpartum^18^. However, these groups were self-selected, and all participants performed regular sustained exercise prior to pregnancy. In contrast, in the present study all the subjects were relatively sedentary at recruitment, which may explain the significant reduction in weight and BMI from pre-pregnancy to follow-up in the exercise group. Thus, although our findings are inconclusive, they suggest a possible beneficial role of exercise in pregnancy in reducing postpartum weight retention and improving later maternal health. Nonetheless, it is not clear whether the observed findings were secondary to exercising during pregnancy or a reflection of ongoing changes in lifestyle after pregnancy.

To the best of our knowledge, this is the first follow-up study to evaluate the effects of antenatal exercise on the offspring up to mid childhood. Indeed, to date only two studies have investigated the effects of maternal exercise on offspring growth and metabolic outcomes. Tanvig *et al*. observed in a follow-up of a RCT no differences in BMI and abdominal circumference in the 2.8-year-old offspring of obese mothers exposed to dietary counselling and physical activity in pregnancy compared to the children of obese controls^26^. In the longer follow-up of a prospective case-control study involving women who were fit prior to pregnancy, Clapp reported that 5-year-old children exposed to antenatal exercise weighed less and had lower adiposity (skinfold thicknesses) than controls^18^. In contrast, although in our RCT there were no differences in anthropometric outcomes at the 1-year follow-up, the offspring of mothers who exercised in pregnancy had more body fat and greater abdominal and gynoid adiposity at the 7-year assessment. The contradictory findings among studies may reflect the different populations studied (i.e. healthy nulliparous and relatively sedentary women in our study compared to very fit and lean women in Clapp’s study or obese women in Tanvig *et al*.)^18,26^. In addition, different times of treatment initiation and exercise prescription were applied, as in Clapp’s study women underwent weight-bearing exercise from before conception throughout pregnancy compared to our design of non-weight-bearing exercise from the second half of pregnancy^18^.

The mechanisms underlying the effects of antenatal exercise on offspring outcomes are unclear. After exercise, intermittent reductions in maternal glucose levels occur, which may lead to adaptations in placental development and function with reduced fetal nutrient supply^19^. In our original trial, the reduced birth size detected in the offspring of mothers who exercised in pregnancy could reflect the repeated effects of maternal exercise^19^. According to the “developmental origins of health and disease” paradigm, early life events are critical for human health and well-being^27^. Many factors influence fetal development, and disturbances in oxygen and nutrients supply may lead to structural and functional changes that can ‘re-program’ the physiology and metabolism of the offspring^27^. Adverse effects in the offspring depend on individual genotype, sex, time of exposure, as well as the type, magnitude, and duration of insult, noting that the timing of onset and the severity of the later metabolic effects also vary^28^. Thus, the increased adiposity observed at 7-year follow-up in children born to exercisers may represent the long-term outcomes of metabolic adaptations that occurred *in utero* in response to moderate-intensity maternal exercise. Maternal ethnicity also has to be taken into account because certain ethnic groups, such as Maori (New Zealand’s indigenous population) and Pacific Islanders, have increased incidences of obesity^29,30^. Similarly, advanced maternal age has been linked to adverse offspring health outcomes such as obesity^31^; conversely, our group has observed increasing parental age at childbirth to be associated with improved offspring metabolism in childhood^32^ and adulthood^33^. Therefore, in the present study both maternal ethnicity and age were included in the multivariable models.

The potential long-term effects of exercise during pregnancy on the offspring have not been properly studied. Nonetheless, there is a widely accepted view that antenatal exercise is safe and should be promoted. The exercise regimen administered in our RCT met current guidelines for pregnant women^4^. Although our findings need to be interpreted with caution due to our limited sample size, they raise questions about the guidelines of many international organisations recommending initiating regular exercise during pregnancy^4,5^. Indeed, our data suggest that long-term offspring health could be affected by the initiation of an exercise program in mid pregnancy. While the limited data from Clapp suggest that continuing preconception exercise throughout pregnancy has long-term beneficial effects on the offspring^18^, this study raises concerns about marked changes in physical activity levels during pregnancy.

The main limitation of our study was the loss (approximately 30%) of participants at the 7-year follow-up, leading to a sample size potentially underpowered to detect statistically significant differences between groups, particularly regarding sex-specific differences in the offspring. In addition, there was a relatively small number of control women assessed at the 1-year follow-up, which would have limited our statistical power at this stage as well. Although our 7-year data indicated a decrease in weight and BMI in mothers who exercised but not among controls, this is not reliable evidence of a treatment effect due to an inflated Type I error rate (i.e. likelihood of wrongly rejecting the null hypothesis)^34^. Further, there are potential limitations associated with the use of self-reported questionnaires to assess maternal and offspring dietary intake and physical activity levels. Nonetheless, this study has a number of strengths. We were able to discard the influence of important potential confounders, such as dietary intake and physical activity levels. Importantly, we provide detailed data on a wide range of metabolic outcomes, and this is so far the largest and longest follow-up study of a RCT of mothers who exercised in pregnancy and their children. Furthermore, the inclusion of relatively sedentary women and the use of non-weight-bearing exercise facilitate the extrapolation of our results to more common populations of pregnant women and their offspring.

In conclusion, our follow-up study provided some evidence of benefit to the mothers approximately 7 years after the RCT. However, maternal physical activity amongst sedentary mothers beginning in the second half of gestation raises questions of potential adverse long-term outcomes in the offspring in mid childhood. Larger studies are required to investigate the effects of the timing and prescription of exercise in pregnancy on the long-term health of the offspring. These should involve a longer follow-up into adolescence, when adiposity amplifies and better predicts the risk of obesity into adulthood.

## Electronic supplementary material

Supplementary Tables

## Data Availability

The clinical data cannot be made available in a public repository due to strict conditions of the ethics approval of our study. Nonetheless, anonymized and unidentifiable data would be made available to other investigators upon request. For this purpose, anyone interested should contact the senior author (P.L.H.).
