# Supplementary material for: Exercise in pregnancy: 1-year and 7-year follow-ups of mothers and offspring after a randomized controlled trial
Source: Sci Rep. 2018 Aug 27;8:12915. doi: 10.1038/s41598-018-30925-5 (PMC6110723; doi:10.1038/s41598-018-30925-5)
Supplement: Supplementary file 1 — Supplementary Tables [file 41598_2018_30925_MOESM1_ESM.pdf]

Exercise in pregnancy: 1-year and 7-year follow-ups of mothers and offspring after a randomized controlled trial

Supplementary Tables

Valentina Chiavaroli[1*], Sarah A Hopkins[1*], José G B Derraik[1,2,3], Janene B Biggs[1], Raquel O Rodrigues[1], Christine H Brennan[1], Sumudu N Seneviratne[1,4], Chelsea Higgins[1], James C Baldi[5], Lesley M E McCowan[6], Wayne S Cutfield[1,2], Paul L Hofman[1†]

[1] Liggins Institute, University of Auckland, Auckland, New Zealand;

[2] A Better Start – National Science Challenge, University of Auckland, Auckland, New Zealand;

[3] Department of Women's and Children's Health, Uppsala University, Uppsala, Sweden.

[4] Department of Paediatrics, Faculty of Medicine, University of Colombo, Sri Lanka;

[5] Department of Medicine, Dunedin School of Medicine, University of Otago, Dunedin, New Zealand;

[6] Department of Obstetrics and Gynaecology, Faculty of Medical and Health Sciences, University of Auckland, Auckland, New Zealand.

* These authors contributed equally to this work.

Supplementary Table 1

Dietary intake and physical activity levels at 7-year follow-up among mothers with completed questionnaires who either exercised in pregnancy or were controls. Data are means ± standard deviations.

		Control	Exercise	p-value
**Dietary intake**	n (proportion of group assessed)	18 (75%)	23 (70%)	
	Energy (kJ)	7255 ± 2472	7114 ± 1849	0.84
	Fat (g)	66 ± 26	70 ± 21	0.59
	Sugar (g)	74 ± 33	81 ± 29	0.46
	Dietary fibre (g)	22 ± 11	23 ± 10	0.83
	Total energy from fat (%)	33 ± 6	35 ± 7	0.32
	Total energy from protein (%)	21 ± 7	19 ± 4	0.33
	Total energy from carbohydrate (%)	42 ± 8	43 ± 9	0.76
**Physical activity**	n (proportion of group assessed)	13 (54%)	22 (67%)	
	Metabolic equivalent of task (MET)	1.66 ± 0.47	1.61 ± 0.44	0.76
	Moderate to high intensity exercise (% MET)	13 ± 11	12 ± 6	0.76

Supplementary Table 2

Dietary intake at 7-year follow-up among children with completed questionnaires and who were born to mothers who exercised in pregnancy or to controls. Age data are means ± standard deviations; other data are means and 95% confidence intervals adjusted for sex and age.

		Control	Exercise	p-value
**n**		18	23	
**Demography**	Age (years)	7.7 ± 0.7	7.5 ± 0.9	0.54
	Sex ratio (males)	39%	78%	**0.022**
	Ethnicity (European)	94%	78%	0.21
**Dietary intake**	Energy (kJ)	7067 (6399–7736)	6598 (5950–7246)	0.12
	Fat (g)	57 (51–65)	50 (44–57)	0.14
	Sugar (g)	89 (72–105)	88 (72–103)	0.93
	Dietary fibre (g)	22 (18–25)	20 (16–23)	0.47
	Total energy from fat (%)	31 (27–34)	28 (25–32)	0.35
	Total energy from protein (%)	15 (14–17)	18 (16–20)	0.06
	Total energy from carbohydrate (%)	52 (48–56)	52 (48–56)	0.98

Supplementary Table 3

Parameters at 7-year follow-up among boys and girls born to mother who exercised in pregnancy or to control mothers, with evidence of sex-specific effects.

		Control	Exercise	p-value
**n**	Boys	10	21	-
	Girls	14	12	-
**Height SDS – MPHSDS** [1]	Boys	1.06 (0.27–1.86)	0.33 (-0.25–0.91)	0.07
	Girls	-0.22 (-0.99–0.55)	0.26 (-0.33–0.85)	0.32
**Diastolic blood pressure (mmHg)** [2]	Boys	55.9 (47.3–64.5)	62.9 (56.8–69.0)	0.13
	Girls	59.8 (54.2–65.5)	56.1 (51.3–60.8)	0.28

MPHSDS, mid-parental height standard deviation score; SDS, standard deviation score.

[1] Means and 95% confidence intervals adjusted for maternal age and ethnicity, and child's age.

[2] Means and 95% confidence intervals adjusted for maternal age and ethnicity, and child's height.
